# WeARTolerance: Evaluating the impact of an arts-based program to reduce mental health-related stigma in young people

**DOI:** 10.1371/journal.pone.0314994

**Published:** 2024-12-31

**Authors:** Ana Beato, Leonor Pereira da Costa, Ana Carvalho, Sara Albuquerque, Isabel Santos

**Affiliations:** HEI‐Lab: Digital Human‐Environment Interaction Labs, Lusófona University, Lisbon, Portugal; University of Malaga: Universidad de Malaga, SPAIN

## Abstract

The stigma surrounding mental health remains a significant barrier to help-seeking and well-being in youth populations. The invisibility of mental health issues highlights the critical need for improved knowledge and stigma reduction, underscoring the urgency of tackling this issue. Arts-based interventions have shown promise in addressing stigma, yet comprehensive longitudinal studies in community settings are limited. This research evaluates the "WeARTolerance’’ arts-based program in reducing mental health stigma among diverse youths. The program integrated psychoeducational and creative activities (e.g., visual arts, cinema, music, and theatre) to explore mental health themes, with 125 teenagers and young adult participants (M = 13.70; SD = 2.06). The present mixed-method study was split into two phases with complementary objectives: 1) evaluate quantitatively the program’s impact in reducing social stigma and related outcomes; 2) explore in-depth qualitative feedback about the program. For the first phase, reliable self-report questionnaires were used to measure mental health knowledge, social stigma, intergroup anxiety, and social distance in pre-, post-, and 6-month follow-up periods. Participants’ age and past psychiatric issues were fixed factors with random intercepts, and mixed effects models were used to analyze the attitudinal outcomes across time. In the second phase of this validation, nine teenagers aged between 12 and 16 participated in two focus groups conducted three months after the program. Its contents underwent thorough analysis using content analysis techniques. The quantitative results from Phase 1 demonstrated a decreasing trend in all primary outcomes. In phase 2, participants acknowledged the activities’ relevance, reported overall satisfaction with the program, and showed great enthusiasm and willingness to learn more. Arts-based interventions like "WeARTolerance" are valuable for challenging stigma and fostering understanding in youth populations and provide an alternative and creative way to increase mental health literacy. The study proposes a program to reduce youth mental health stigma through arts-based elements, early intervention, and psychoeducation, involving collaborations between professionals and artists to promote youth engagement. Future studies should include indirect social contact and randomized controlled interventions.

## Introduction

Mental health problems are a leading global cause of disability and morbidity [1], affecting around 22% of the Portuguese population. Particularly concerning is the situation among adolescents aged 10–19, with one in seven experiencing a mental health problem. The recent 2021/22 HBSC study highlights a troubling decline in the mental well-being of Portuguese adolescents, marked by rising rates of unhappiness, self-harm, and various psychological and physical symptoms. These statistics emphasize the urgent need for effective interventions to address this growing public health concern. Untreated mental health issues in youth can lead to severe consequences, including reduced educational opportunities, limited healthcare access, increased victimization, unemployment, poverty, and heightened internalized stigma [2, 3]. The long-term effects of untreated mental health conditions can manifest as significant social, physical, and work-related problems in adulthood [4, 5]. Also, public sector spending on untreated young people with mental health conditions is ten times higher than for those without, creating a substantial economic burden [6].

One of the major barriers to addressing adolescent mental health effectively is the significant delay between the onset of symptoms and seeking treatment [7]. A nationally representative study revealed that 65.4% of Portuguese individuals meeting criteria for a past-year mental disorder did not use mental health services, despite universal healthcare access [8], with stigma being identified by the World Health Organization [9] as a crucial barrier in this context. Next, we will explore the significant impact of mental-health-related stigma on treatment-seeking and health outcomes, the theoretical frameworks explaining stigma, and the potential of arts-based interventions to address these issues.

Stigma impacts help-seeking behavior [10–12] by creating a social environment where individuals with mental health problems are not only discouraged from seeking help but also face reduced healthcare access and increased socioeconomic burden [13]. Evidence also shows that stigma harms healthy behaviors (e.g., diet and exercise) and encourages risky behaviors (e.g., substance abuse), which contributes to resistance to healthcare, poorer health outcomes, and higher morbidity and mortality [10, 14].

To address stigma, it is essential to examine the theoretical frameworks that explain its effects. The social cognitive model explains stigma through stereotypes (negative beliefs), prejudice (cognitive and emotional reactions), and discrimination (behavioral responses) [13–16]. Labeling theory [15] highlights stigma arising from labeling, stereotyping, and discrimination in power situations, with manifestations at individual, interpersonal, and sociocultural levels [11]. Stigma is categorized into social stigma (public attitudes), self-stigma (internalized beliefs), and structural stigma (discriminatory policies) [14–17]. Social stigma notably impacts mental health by reducing resource investment, leading to inadequate care, income loss, unemployment, limited housing access, and diminished social support [10, 13].

Mental-health-related stigma often arises from a lack of knowledge or misconceptions about mental health [16]. According to Attribution Theory [18], individuals may attribute mental health problems to controllable factors (e.g., perceived lack of effort), which leads to negative emotional reactions and behavioral responses, including increased social distance from those affected [19]. Such social distance from others is associated with a reduced willingness to seek professional help [20]. Furthermore, Intergroup Anxiety, which includes affective, cognitive, and physiological components, exacerbates stigma by making anticipated interactions with individuals with mental health problems stressful and undesirable [21, 22].

In Portugal, a randomized controlled trial involving university students to reduce depression stigma showed direct effects of better help-seeking behaviors [23]. Interventions to address stigma are not only promising but crucial, especially for adolescents whose self-concept is heavily influenced by peer opinions [24]. Young people generally have low mental health literacy and hold more negative and stigmatizing attitudes toward mental health problems, often viewing them as personal failures, due to social pressure and fear of being labeled [25]. Early implementation of anti-stigma education to increase awareness and knowledge of mental health can encourage young people’s timely care-seeking and promote respect, diversity, and social inclusion for all [26].

The knowledge-attitude-behavior paradigm has been employed in designing stigma-reducing interventions [27, 28], which often include educational and social contact components [17, 29]. Educational interventions aim to enhance mental health literacy, while social contact interventions facilitate direct interactions between participants and individuals with mental health problems [16]. Research, including a meta-analysis by Corrigan et al. [17], indicates that both types of interventions can positively impact stigma, attitudes, and behavioral intentions, with educational interventions proving particularly effective in changing adolescents’ attitudes.

Arts-based interventions have recently gained attention as a promising approach for stigma reduction among youth. These interventions, which include visual, literacy, and performing arts, are designed to engage participants creatively and expressively, potentially offering a holistic means of addressing mental-health-related stigma [27]. Participatory arts-based interventions (PAB), which promote community engagement through activities, such as murals, theatre workshops, and art therapy sessions, are particularly noteworthy for their non-clinical, collaborative, and flexible nature, directed at all adolescents with and without mental health problems, and the potential to foster personal growth and social change [30–32]. Despite their promise, there is a need for more rigorous and longitudinal research to assess efficacy thoroughly, especially in community samples of adolescents and young adults.

### Current study

Our study aims to evaluate the impact of an arts-based program on reducing mental-health-related stigma among youth, by employing a within-subjects design with multiple measurement moments. This mixed-methods study will be carried out in two phases. Phase 1 involves analyzing changes in participants’ mental health knowledge, social stigma, intergroup anxiety, and social distance both immediately after the program and again six months later. In Phase 2, qualitative feedback will be collected to gain insights into participants’ experiences, engagement, satisfaction, and perceptions of the program’s effectiveness in reducing mental health-related stigma.

There is an urgent need to tackle the pervasive issue of mental-health-related stigma, which significantly hinders effective treatment and exacerbates negative outcomes associated with mental health problems [33]. Traditional interventions have proven effective, yet there is growing recognition of the potential for arts-based approaches to offer a more engaging and comprehensive method for addressing stigma [34]. By involving youths in creative activities that resonate with their developmental needs and social contexts, arts-based interventions provide unique opportunities for reducing stigma, improving mental health literacy, and fostering positive attitudes and behaviors towards mental health [35].

This study seeks to fill a critical research gap, offering valuable insights into the efficacy of creative methods and informing the development of more effective, youth-centered interventions to decrease mental health stigma, which can potentially enhance mental health outcomes.

## Materials and methods

### Participants

The inclusion criteria involved being 11 to 24 years old, speaking Portuguese, living in Portugal, and being willing to participate in an arts-based program. For participants under 18, it was also a criterion to have their legal guardian’s formal authorization to participate. On the other hand, the exclusion criteria included significant cognitive, motor, and speech impairments that might hinder the completion of the activities. This information was provided in the informed consent provided to the parents when participants were minors, with a clear statement that these limitations could be difficult for participation in the program´s activities and/or the completion of the research protocol.

**Fig 1:**
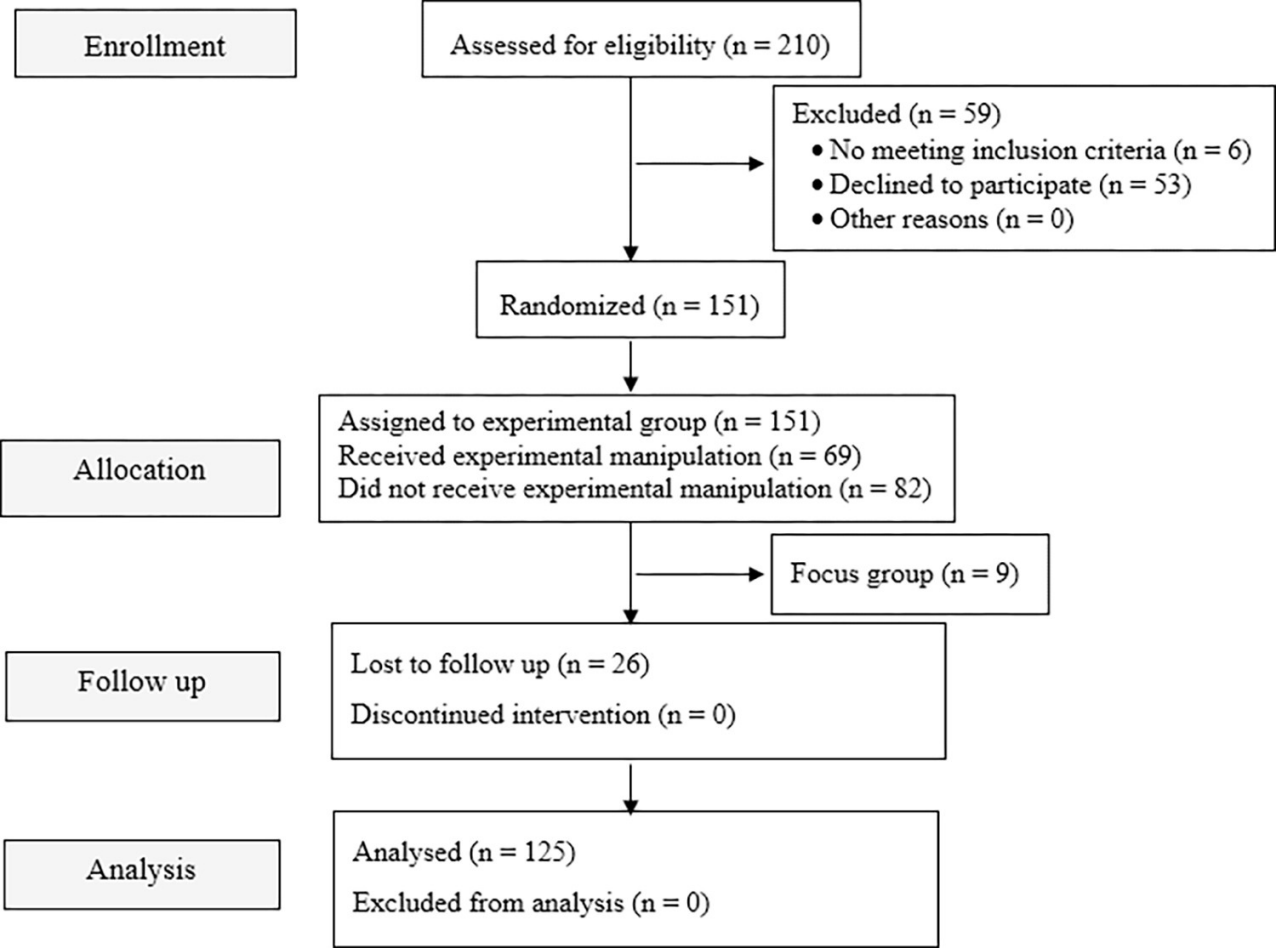
Consort diagram.

Power analysis for repeated measures ANOVA was conducted in G*Power 3.1 [36] with the following parameters: one group, four measurements, a power of .95 and alpha of .05, a small effect size (*f* = .15), a moderate correlation between repeated measures (*r* = .50), establishing a minimum sample size of 97. Fig 1 illustrates the flow of participants during the different phases of the present study.

### Design and procedures

This study uses a mixed-method explanatory nested design [37], combining qualitative and quantitative methods to explore the intervention’s efficacy. In addition to the primary quantitative aim of evaluating the program’s efficacy, a complementary aim was embedded within the study to qualitatively assess participants’ in-depth perceptions and satisfaction after the intervention.

This research, approved by the Ethics and Deontology Committee of the Psychology and Life Sciences at Lusófona University, was conducted with a strong commitment to ethical standards. Before enrollment, participants received accurate information regarding the features and goals of the study. They were informed about the voluntary nature of their involvement and that they could withdraw at any moment. When participants experienced discomfort during the program, the psychological team offered support. In addition, participants were given a list of public psychological services if they needed them.

Participants were recruited using two sampling methods (snowball and convenience) in public and private schools and universities and through institutions of the community and services directed to mental health and neurodiversity. Flyers and social media were also used to recruit youth from the larger community. All adult participants provided their written consent to participate. For minor participants, legal guardians provided written consent, authorizing their participation, and minors also provided their written consent to participate. Consents were obtained at all phases of the study through a link and the assignment of a code to match responses while maintaining anonymity. After recruitment, participants were randomly assigned to one of eight smaller groups according to their age and developmental level (teenagers: 11–16 years old vs. young adults: 17–24 years old). Although the participants were assigned to the conditions in a randomized way, the team guaranteed a similar proportion of youngsters with mental health conditions within all groups.

Quantitative data was collected at three points through a structured questionnaire hosted on Qualtrics. The pre-test was administered to all participants the week before the program (T1: baseline), the post-test a week after the program (T2: post-intervention), and the follow-up six months after the program (T3: follow-up). The program was scheduled during official school vacations due to its intensive and short-term nature. The participants were randomly allocated into four groups that were assessed in three moments. A matching technique was used to ensure the groups had similar key characteristics before the assignment. This method increases internal validity by reducing the risk of confounding variables affecting the outcome by balancing participants across groups based on specific variables (i.e., gender, age, and presence/absence of mental health problems) and ensuring differences between groups are due to the intervention, not demographic disparities [38]. Qualitative data was collected through Focus Groups (FG). The FG procedure, designed to gather participants’ perspectives on the program, involved randomly selected participants being invited to share their opinions. Each session, lasting 60 minutes, was facilitated by two researchers from the team. Two focus group sessions were conducted, with a total of nine participants aged 12 to 16 years (M = 13.78; SD = 1.56), 55.56% female. The FG sessions were video recorded with the proper authorization from the participants and, in the case of minors, from their guardians. Subsequently, the content of the interviews was transcribed for further analysis. To evaluate the program qualitatively, a structured guide was developed, covering different domains: Pre-program Expectations (e.g., participants were asked whether they joined voluntarily and what they expected to find before the program started); Program Operation (e.g., the discussion explored participants’ thoughts on the timing of the program, its duration, memorable themes or activities, and their opinions on the facilitators and overall structure); Benefits and Positive Aspects (e.g., participants were encouraged to share what they liked most about the program, including any surprises or positive experiences); Negative Aspects (e.g., the discussion also addressed what participants liked least about the program and what improvements they would suggest if the program were to have future editions); Threats (e.g., participants were asked to identify any factors they believed compromised or hindered the program’s execution); Opportunities (e.g., the conversation also focused on what participants felt facilitated the program’s smooth operation and contributed to its success); Lessons Learned (e.g., participants were invited to reflect on any new knowledge or insights they gained, particularly regarding mental health-related stigma); Post-program Attitudes (e.g. the discussion explored whether there had been any changes in the participants’ views on people with mental health problems since attending the program and how the program influenced their attitudes or behaviors) and; Final Opinions and Suggestions (e.g., finally, participants shared their thoughts on how the arts can raise awareness and address important themes like stigma and mental health. They also offered suggestions on how the program could evolve if it continued and whether they would recommend it to a friend, along with their reasons). This structured approach, covering a wide range of domains, ensured a comprehensive program evaluation from the participants’ perspectives, providing valuable insights for future improvements.

Table 1 presents the organization, timing, and groups included in the moments of the intervention and their respective evaluations.

**Table 1:** Phases of the intervention program.

Group	T1	Program	T2	Focus groups	T3
1	27th of March until 3rd, April, 2023	3rd–6th, April, 2023	6th–13th, April, 2023	September 8, 2023	October, 2023
2	4th–11th of April, 2023	-	11–15 April, 2023	-	October, 2023
3	12th–19th of June, 2023	19th–22nd of June, 2023	22nd–29th of June, 2023	September 8, 2023	December, 2023
4	19th–26th of June, 2023	-	29th June-6th of July, 2023	-	December, 2023

### Program description

**Fig 2:**
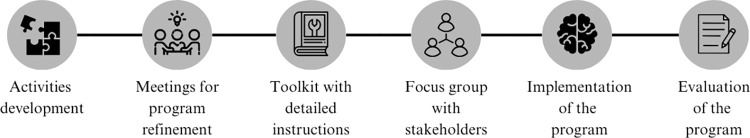
Graphical abstract regarding program development.

The program’s development lay in the collaborative efforts between a psychology team and educators representing diverse artistic disciplines, such as visual arts, theatre, cinema, and music, ensuring a multifaceted approach that catered to varied learning styles. Artistic expression allows adolescents to explore their thoughts and feelings nonverbally, empowering them to challenge stigmatizing beliefs. This PAB program fosters community and belonging, fostering open mental health and stigma dialogue. Key steps in its development included: 1) activities that leverage the unique expressive qualities of each art form, fostering discussions about mental health and stigma; 2) systematic meetings to refine objectives and content that align with overarching goals and consider the sensitivity of the topics covered; 3) preparation of a session-by-session manual (Toolkit) with detailed instructions to guide facilitators; and 4) focus-group assessment with specialists and stakeholders before implementation to provide feedback on content and delivery methods, ensuring authenticity and responsiveness to the target audience needs. This approach ensured the program was well-structured, inclusive, and effective in addressing mental health-related stigma. Fig 2 portrays the process of program development.

**Fig 3:**
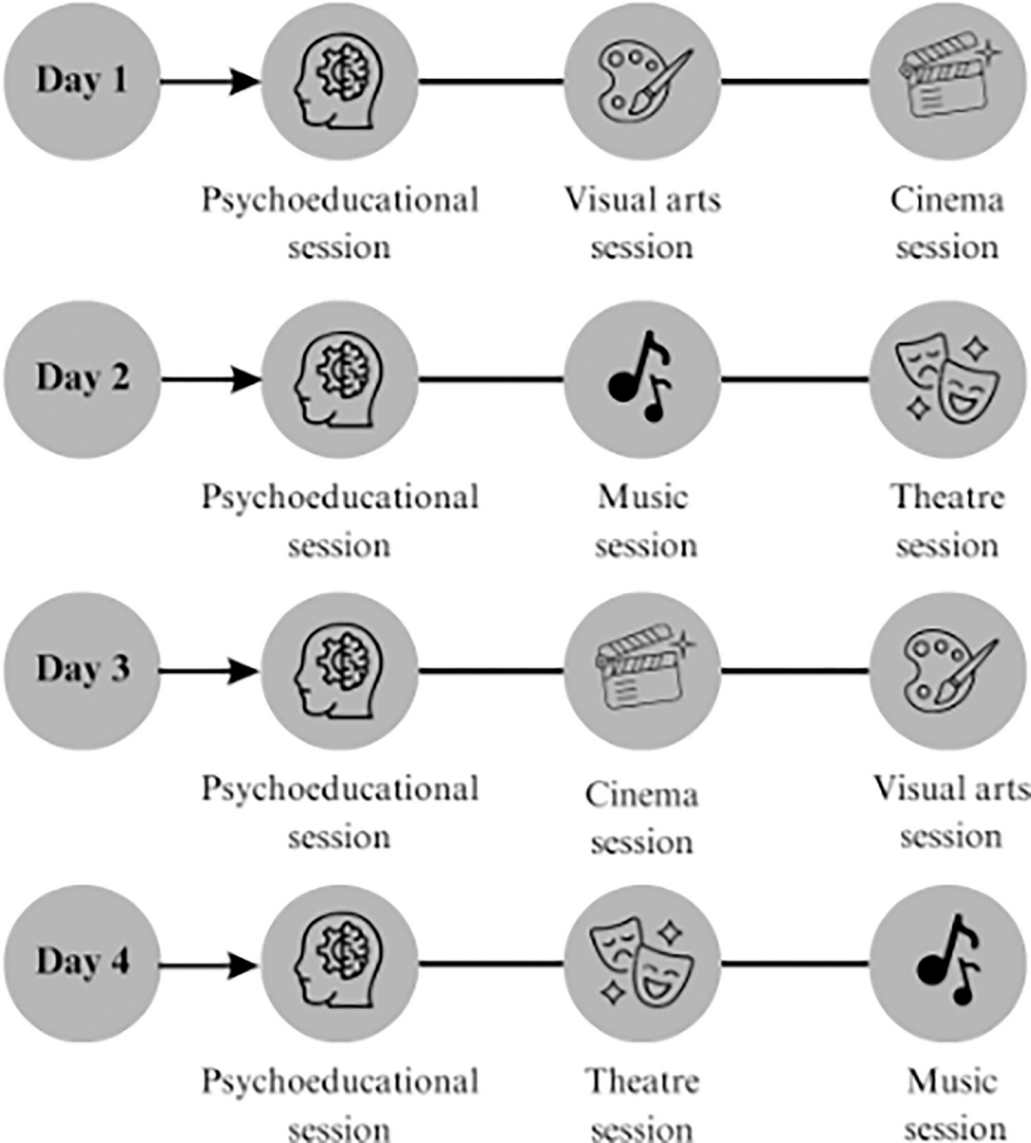
Graphical abstract regarding the program organization.

The program consisted of 12 sessions over four sequential full days. Each of the four days started with a psychoeducational session led by the psychology team, followed by two sessions among visual arts, cinema, music, and theatre, one in the morning and the other in the afternoon. afternoon. Fig 3 illustrates the organization of the program’s sessions during its four days, although the sequence of the art sessions was randomized according to the group of participants.

Table 2 presents an overview of the WeARTolerance program and details the focus of each session.

**Table 2:** Overview of the WeARTolerance program.

Art/ Session	The focus of the session
Psychology	
Session 1	Welcome session. Individual reflection on expectations of the program, icebreaker exercise, and individual reflection on mental health and stigma. Group dynamics and exploration of knowledge and attitudes.
Session 2	Icebreaker exercise. Psychoeducation uses a video about talking about mental health. Psychoeducation uses testimonies from public figures with a history of mental illness.
Session 3	Relaxation and mindfulness. Psychoeducation and group reflection after watching a video about stigma and mental illness. Group dynamics.
Session 4	Relaxation, emotional regulation, and compassion. Psychoeducation and individual reflection using a video about talking about perfection not existing. Psychoeducation and teamwork after watching a video about testimonies from people who talk about their mental illness and another video about breaking down the stigma of mental illness. Closing session.
Theatre	
Session 1	Facilitating integration into the group and promoting socio-emotional skills
Session 2	Promoting emotional awareness, empathy skills, and acceptance of difference
Music	
Session 1	Raising participants’ awareness, validation, appreciation, and acceptance of difference.
Session 2	Learning to communicate through music.
Visual Arts	
Session 1	Promoting the acceptance of difference and diversity through observing, manipulating, and describing concrete physical objects.
Session 2	Building a self-representation from silhouettes and symbolic objects. Building a collective representation means understanding that there are many and sometimes unexpected points of unity between the various participants in the group.
Cinema	
Session 1	Recording a film scene related to a story about a mental health problem was selected during the session.
Session 2	Acquire editing/editing skills for the recording made in the film production studio, considering the theme of mental health.

The psychoeducational sessions included activities that promote relaxation and mindfulness, emotional regulation, and compassion. These were followed by informative videos or presentations about mental health and stigma and activities encouraging sharing, reflection, and discussion of participants’ understandings and experiences.

For the arts-based activities, the participants were divided into two smaller groups (12–15 participants), and the order of the art sessions in each group was randomized. Theatre activities (e.g., role-play, role-taking, improvisation) aimed at stimulating imagination, empathy, and understanding of scenic space, and using the body as a language of improvisation to enhance the ability to respond to unfamiliar behaviors and interactions. Cinema activities, such as production, directing, and acting, focused on developing key skills like teamwork, planning, decision-making, mental flexibility, and openness to diverse perspectives. Visual arts activities, including painting self-portraits and personal objects or interpreting silhouettes and shadows, aimed to explore personal symbolism and highlight both the differences and similarities among participants, fostering a sense of interconnectedness and collaboration. Finally, music sessions, which included sensory exercises and improvisation, were designed to cultivate teamwork, respect for others’ ideas, and collective music creation. These sessions also aimed to promote tolerance, respect, and openness, while helping to reduce the social stigma associated with mental health.

### Measures

The research protocol included questions about sociodemographic characteristics: age, gender, school grade, level of education, nationality, perceived social status, household, diagnosis of mental health and neurodevelopmental problems, taking psychiatric medication, having specialized assistance for mental health problems, and contact with people with mental health and neurodevelopmental problems. If the participants were minors, their parents answered these questions in a brief questionnaire provided after the informed consent form. Notably, the following self-report instruments to measure the primary outcomes were used:

1. *Knowledge on mental health*: Portuguese Version of Mental Health Knowledge Schedule (MAKS) [39, 40], which consists of six items aimed at measuring knowledge about mental health stigma (e.g., *“People with severe mental health problems can fully recover*.*")* and six items aimed at measuring ability to identify mental health problems (e.g., *“Depression”*), rated on a 5-point Likert scale (*1 = strongly disagree* to *5 = strongly agree*). Items 6, 8, and 12 are reversed-coded. The scale score is obtained by summing the items, with a higher score indicating more excellent knowledge about mental health stigma. The present study obtained a Cronbach’s alpha of 0.57 in the pre-test, 0.62 in the post-test, and 0.28 in the follow-up. The low Cronbach’s alpha scores, particularly in the follow-up, can likely be attributed to the fact that the MAKS was not originally designed to function as a scale, making internal consistency less relevant in this context [39]. The MAKS scale intentionally includes items with a multidimensional structure to assess various types of mental health knowledge. Consequently, high internal consistency is not expected, as individuals may possess knowledge in certain domains but lack it in others, as occurred in the validation of the original scale (α = 0.65; [39]) and in the validation of the Portuguese translation (α = 0.29; [40]). As such, we chose to proceed with the analysis and use the scale as is, given that it has been extensively studied and validated in this domain.
2. *Social distance*: The Social Distance Scale [41, 42], translated into Portuguese within the scope of this study, comprises eight items related to closeness towards people with mental health problems (e.g., *“Would have as my regular friends*.*"*), rated on a 4-point Likert scale (1 = I would like it a lot to 4 = I wouldn’t like it). The scale score is obtained by averaging the items, with a higher score associated with greater social distance. The present study obtained a Cronbach’s alpha of 0.93 in the pre-test, 0.93 in the post-test, and 0.89 in the follow-up.
3. *Intergroup anxiety*: Intergroup Anxiety Scale [43], translated into Portuguese language within the scope of this study, which consists of seven items regarding emotions towards people with mental health problems (e.g., *“Anxious”*), rated on a 7-point scale (*1 = I wouldn’t feel like that at all* to *7 = I would feel like that*). Items 3, 4, and 7 are reversed-coded. The scale score is obtained by summing the items, with a higher score indicating greater intergroup anxiety. The present study obtained a Cronbach’s alpha of 0.72 in the pre-test, 0.76 in the post-test, and 0.74 in the follow-up.
4. *Social stigma*: Portuguese Version of Attribution Questionnaire AQ-9 for adults [44, 45] and Portuguese Version of AQ-8-C for children and adolescents [46, 47]. The AQ-9 comprises nine items, and the AQ-8-C comprises eight items aimed at measuring social or public stigma towards people with mental illnesses (e.g., *“How scared of José would you feel*?*’*), rated on a 9-point Likert scale (*1 = no or nothing to 9 = very much or completely*). In both instruments, item 7 is reversed-coded, and the total score is obtained by averaging the items, with a higher score indicating greater social stigma. In the present study, AQ-9 obtained a Cronbach’s alpha of 0.86 in the pre-test and 0.71 in the post-test, while AQ-8-C obtained a Cronbach’s alpha of 0.25 in the pre-test, 0.58 in the post-test, and 0.64 in the follow-up. Regarding Cronbach’s alpha of AQ-9 in the follow-up, it was impossible to calculate it due to the low number of responses from adult participants (*n* = 2) at this moment of data collection. The low Cronbach’s alpha observed at the initial phase of the longitudinal study for the AQ-8-C can be attributed to its multidimensional nature, where each item measures a different aspect of stigma (e.g., blame, pity). As the study progressed, alpha values improved, which may be due to increased familiarity with the scale and enhanced understanding of mental health stigma following an intervention. We chose to proceed with the analysis and use the scale as is, given that it has been extensively studied and validated in this domain.
5. *Focus-group script*: Open-ended questions were prepared to explore participants’ perspectives (e.g., “*What did you think about the inclusion of activities from these four art forms*?*”*), experiences (e.g., “*What did you learn in the program*?*”*), engagement with (e.g., *“Did the program contribute to any change in the way you perceive people with mental health problems*?*”*), satisfaction (e.g., *“What did you like the most about the program*?*”*), perceived benefits (e.g., *“To what extent was the program helpful in changing your attitude or behavior towards this issue*?*”*) or drawbacks (e.g., *“If there were more editions of the program*, *what do you think can be improved*?*”*. The script is available at https://osf.io/hwrka/?view_only=b52e2475c9b145eda84d140188ee8102.

### Data analysis

Four linear mixed-effects models were conducted, combining both fixed and random predictor variables, to account for the interdependent nature of the data (e.g., repeated measures pre-program, post-program, and follow-up). As dependent variables, we used the four attitudinal outcomes measured across time: the Mental Health Knowledge Scale (MAKS), the Social Distance Scale (SDS), the Intergroup Anxiety Scale (IAS), and the Attribution Questionnaire (AQ). Time was included as a fixed factor rather than a random one to test the effect of time directly on the scores of each outcome measure. The main effects of age and previous psychological problems (0 = no, 1 = yes) were included as fixed covariates. For the random effects, we included random intercepts for participants to adjust for possible variation at each participant’s baseline.

The analyses were conducted in R statistical software, version 4.2.1 (R Core Team, 2022), using the lme4 package [48, 49] to estimate the linear mixed effects models. The models were fitted using Restricted maximum likelihood (REML), and p-values were obtained using the Satterthwaite approximation [50] via lmerTest package [51]. First, Time was used as a numeric variable to test the overall trend of scores over time. Afterward, pairwise comparisons were conducted to calculate the significant differences in scores between each pair of time points (Baseline vs. Post-Intervention, Post-Intervention vs. Follow-Up, and Baseline vs. Follow-Up). This approach allows us to assess if the overall findings hold across different time points and whether the strength of the effects changes over time. The significance level used in the study was α = 0.05. This threshold was applied to determine statistical significance for all hypothesis tests conducted.

Regarding the qualitative evaluation of the program, two focus groups involving program participants were conducted by two experienced psychologists and an observer. The data obtained were transcribed and analyzed using deductive content analysis in Microsoft Excel. Initially, one researcher (AC) read the transcribed data to get an overall sense of the content and analyzed it using the question structure previously employed during the focus groups. Based on these questions, the researcher defined the main categories and subcategories to highlight the most relevant aspects for the qualitative evaluation of the program. Then, the researcher coded the participants’ responses and organized the data according to the categories and subcategories previously defined. Following this, another researcher (AB) meticulously examined the transcripts, which enabled the refinement of the categorization to reflect the participants’ responses accurately. The researchers (AC and AB) discussed the categories until a consensus was reached regarding the results.

## Results

The final sample consisted of 125 participants from 11 to 21 years old (*M* = 13.70, *SD* = 2.06). From this sample, nine participants aged between 12 and 16 (M = 13.8; SD = 1.6), of whom 55.6% were female, participated in focus groups to qualitatively evaluate the program. Table 3 presents the sociodemographic characteristics of the sample.

**Table 3:** Characteristics of the sample.

Sociodemographic characteristics	Sample number/proportion N (%)
Gender	
Female	78 (63.9%)
Male	44 (36.1%)
Nationality	
Portuguese	116 (94.3%)
Other	7 (5.7%)
Mental health and neurodevelopmental problems	
Yes	17 (13.6%)
No	108 (86.4%)
Psychiatric medication	
Yes	11 (64.7%)
No	6 (35.3%)
Live with someone with mental health and neurodevelopmental health problems	
Yes	23 (18.7%)
No	100 (81.3%)

Regarding their perceived social status (MacArthur Scale of subjective social status), participants ranked themselves above the midpoint of the scale (*M* = 7.01, *SD* = 1.66), meaning that they feel they have a moderate level of social status compared to others in their community or society. Regarding mental health and neurodevelopmental problems, 75% (*n* = 12) of participants with problems reported experiencing anxiety disorders, 37.5% (*n* = 6) reported attention deficit hyperactivity disorder (ADHD), and 31.3% (*n* = 5) reported mood disorders (e.g., depression).

### Phase 1: Quantitative evaluation of the intervention

The full dataset with all coded variables is available as Data S1 via the link https://osf.io/hwrka/?view_only=b52e2475c9b145eda84d140188ee8102. Table 4 presents the descriptive statistics for the four outcome measures in the three-time evaluation points, and the estimates of the linear mixed effects models for the outcomes presented above.

**Table 4:** Descriptive statistics of outcomes at baseline, post-intervention, and follow-up and estimates of the linear mixed effects model for the outcomes.

	Mental Health Knowledge				Social Distance				Intergroup Anxiety				Social Stigma			
Descriptives	*n*	*M*	*SD*		*n*	*M*	*SD*		*n*	*M*	*SD*		*n*	*M*	*SD*	
Baseline	125	41.19	7.77		125	2.17	0.61		125	21.92	7.33		125	3.33	0.88	
Pos-Intervention	125	44.00	6.43		125	1.95	0.57		125	19.62	7.40		125	2.37	0.95	
Follow-up	76	43.74	5.04		76	2.05	0.43		76	19.57	6.60		76	2.49	0.96	
**Liner Mixed Effects Model**																
**Fixed Effects**	***b***	***SE***	***t***	***p***	***b***	***SE***	***t***	***p***	***b***	***SE***	***t***	***p***	***b***	***SE***	***t***	***p***
Intercept	34.50	4.68	7.37	< 0.001	2.29	0.41	5.58	< 0.001	21.43	4.99	4.29	< 0.001	5.02	0.67	7.50	< 0.001
Time	1.23	0.39	3.17	0.002	- 0.07	0.03	- 2.27	0.024	- 1.02	0.39	- 2.64	0.009	- 0.48	0.05	- 9.31	< 0.001
Age	0.31	0.24	1.29	0.201	- 0.02	0.02	- 1.09	0.278	0.50	0.26	1.96	0.052	- 0.07	0.03	- 1.93	0.057
Psych. Problems	- 0.94	1.44	- 0.65	0.516	- 0.12	0.13	- 0.92	0.362	- 3.11	1.54	- 2.02	0.045	- 0.25	0.21	- 1.21	0.230
**Random Effects**	***σ2***	***SD***			***σ2***	***SD***			***σ2***	***SD***			***σ2***	***SD***		
ID (Intercept)	18.8	4.34			0.17	0.41			23.04	4.80			0.42	0.65		
ICC	0.397				0.595				0.459				0.464			
**Model Fit**	**AIC**	**BIC**			**AIC**	**BIC**			**AIC**	**BIC**			**AIC**	**BIC**		
	2140	2163			507	530			2152	2175			856	879		

#### Mental health knowledge

The analysis examining the overall trend of MAKS over time revealed a positive main effect of Time (*b* = 1.23, *SE* = 0.39, *t* (205.91) = 3.17, *p* = .002, 95% CI [0.47, 2.00]), showing that knowledge regarding mental health issues improved over time.

**Fig 4:**
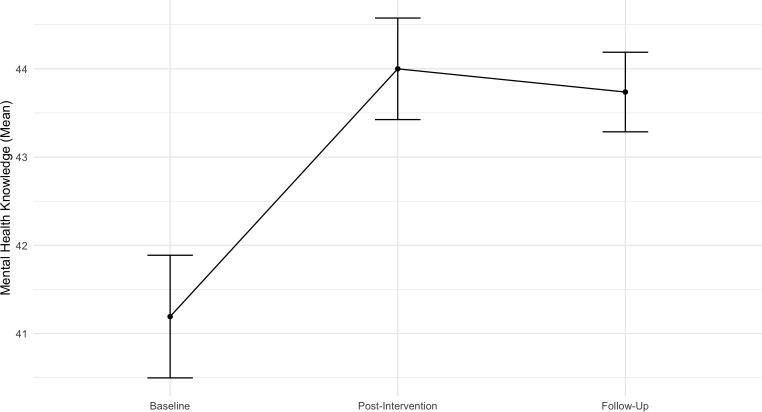
Means with standard errors across time points for the outcome of mental health knowledge.

Contrasts analysis revealed significant differences between time points, namely a significant increase in participants’ knowledge of mental health issues from pre-intervention to immediately after the intervention (*b* = 2.81, *SE* = 0.648, *p* < .001) and to the follow-up (*b* = 1.99, *SE* = 0.773, *p* = .029). As such, this increase was maintained at the follow-up assessment as there was no significant difference between post-intervention and follow-up time points (b = - 0.81, SE = 0.773, p = .885). Age (*b* = 0.31, SE = 0.24, t (110.77) = 1.29, p = .201, 95% CI [− 0.16, 0.78]) and previous psychological problems (*b* = - 0.94, SE = 1.44, t (110.30) = - 0.65, *p* = .516, 95% CI [− 3.75, 1.87]) were not statistically significant. Fig 4 illustrates means and standard errors across time for this outcome.

The random intercept model indicated significant variability in individuals’ knowledge about mental health issues (*p* < .001). The estimated variance component for the random intercept was 18.8 (*SD* = 4.34), suggesting substantial variability in baseline levels of the outcome across individuals. Approximately 41% of the total variance in the outcome is attributable to between-individual differences (ICC = 0.405).

#### Social distance

**Fig 5:**
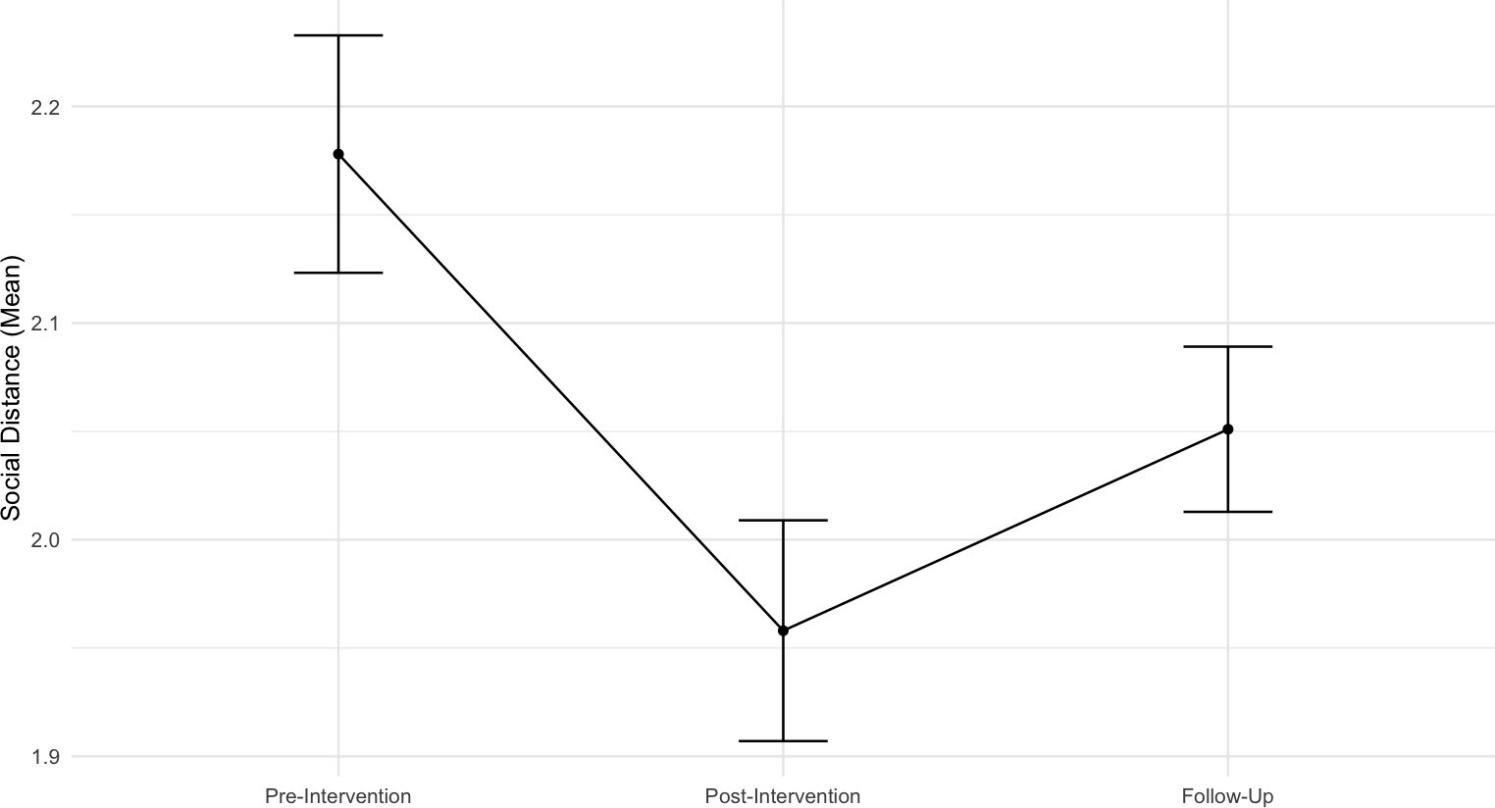
Means with standard errors across time points for the outcome of social distance.

The linear mixed effects model examining the overall trend of social distance over time revealed a negative effect of Time (*b* = - 0.07, *SE* = 0.03, *t* (207.80) = - 2.27, *p* < .001, 95% CI [- 0.12, - 0.01]), showing that over time participants showed a greater willingness to get closer to people with mental health problems. Contrast analysis revealed significant differences between time points. Specifically, results showed a significant decrease in participants social distance issues from pre-intervention to immediately after the intervention (*b* = - 0.22, *SE* = 0.048, *p* < .001), but this did not maintain significance to the follow-up (*b* = - 0.09, *SE* = 0.057, *p* = .394). Differences between post-program and follow-up are marginally significant (*b* = 0.13, *SE* = 0.057, *p* = .056), showing a slight increase. Age (*b* = - 0.023, *SE* = 0.02, t (117.44) = - 1.09, *p* = .278, 95% CI [− 0.06, 0.01]) and previous psychological problems (*b* = - 0.12, SE = 0.13, t (117.09) = - 0.92, *p* = .362, 95% CI [− 0.36, 0.13]) were not statistically significant. Fig 5 illustrates means and standard errors across time for this outcome.

The random intercept model showed significant variability between individuals in their desire to maintain less distance from people with mental health issues (*p* < .001), with an estimated variance component of 0.17 (*SD* = 0.41) and an ICC of 0.525, indicating that 53% of the total variance was due to between-individual differences.

#### Intergroup anxiety

**Fig 6:**
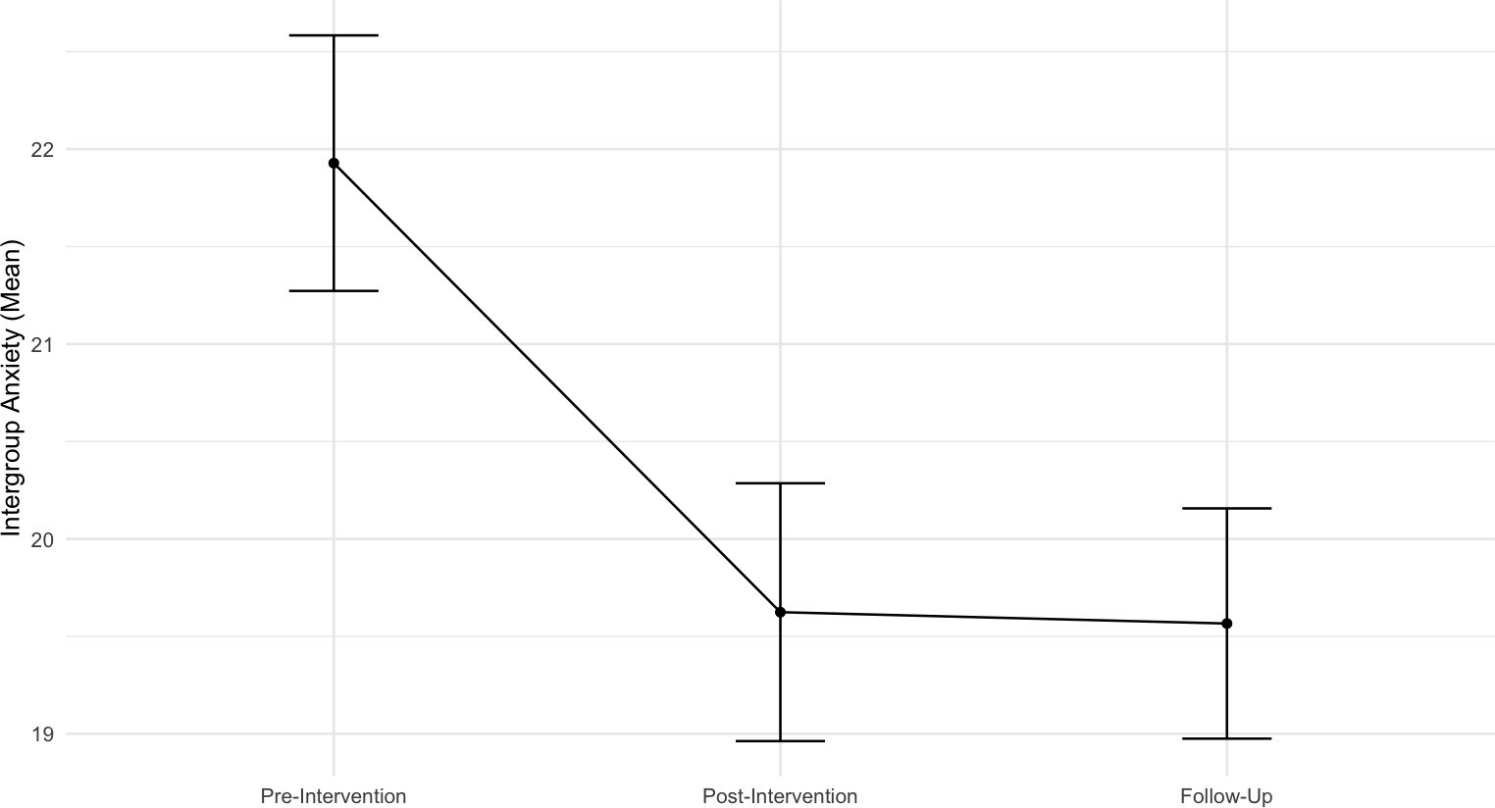
Means with standard errors across time points for the outcome of intergroup anxiety.

The results of the linear mixed effects for intergroup anxiety revealed a significant negative effect of time (*b* = − 1.02, SE = 0.39, t (210.41) = − 2.64, *p* = .009, 95% CI [− 1.78, − 0.26]), indicating that participants exhibited less intergroup anxiety with individuals facing mental health challenges as time advanced. Contrast analysis results showed a significant decrease in participants intergroup anxiety levels from pre-intervention to immediately after the intervention (*b* = - 2.30, *SE* = 0.649, *p* < .001), but this did maintain significance to the follow-up (*b* = - 1.66, *SE* = 0.776, *p* = .098). There were no differences between post-program and follow-up (*b* = 0.65, *SE* = 0.776, *p* = 1.00). Age (*b* = 0.50, *SE* = 0.26, *t* (117.53) = 1.96, *p* = 052, 95% CI [0.002, 1.00]) was marginally significant, showing that with age, there is a tendency to face more anxiety about people with mental problems. The variable Previous psychological problems (*b* = 3.11, *SE* = 1.54, *t* (117.11) = 2.02, *p* = .045, 95% CI [0.11, 6.10]) are positively associated with intergroup anxiety, showing that participants who have previous psychological problems face overall higher intergroup anxiety than those who do not have previous psychological problems. Fig 6 illustrates the means and standard errors across time for this outcome.

The random intercept model indicated significant variability between individuals in their anxiety related to people with mental health issues (p < .001), with a variance component of 23.04 (SD = 4.80) and an ICC of 0.459, suggesting 46% of the variance was due to between-individual differences.

#### Social stigma

**Fig 7:**
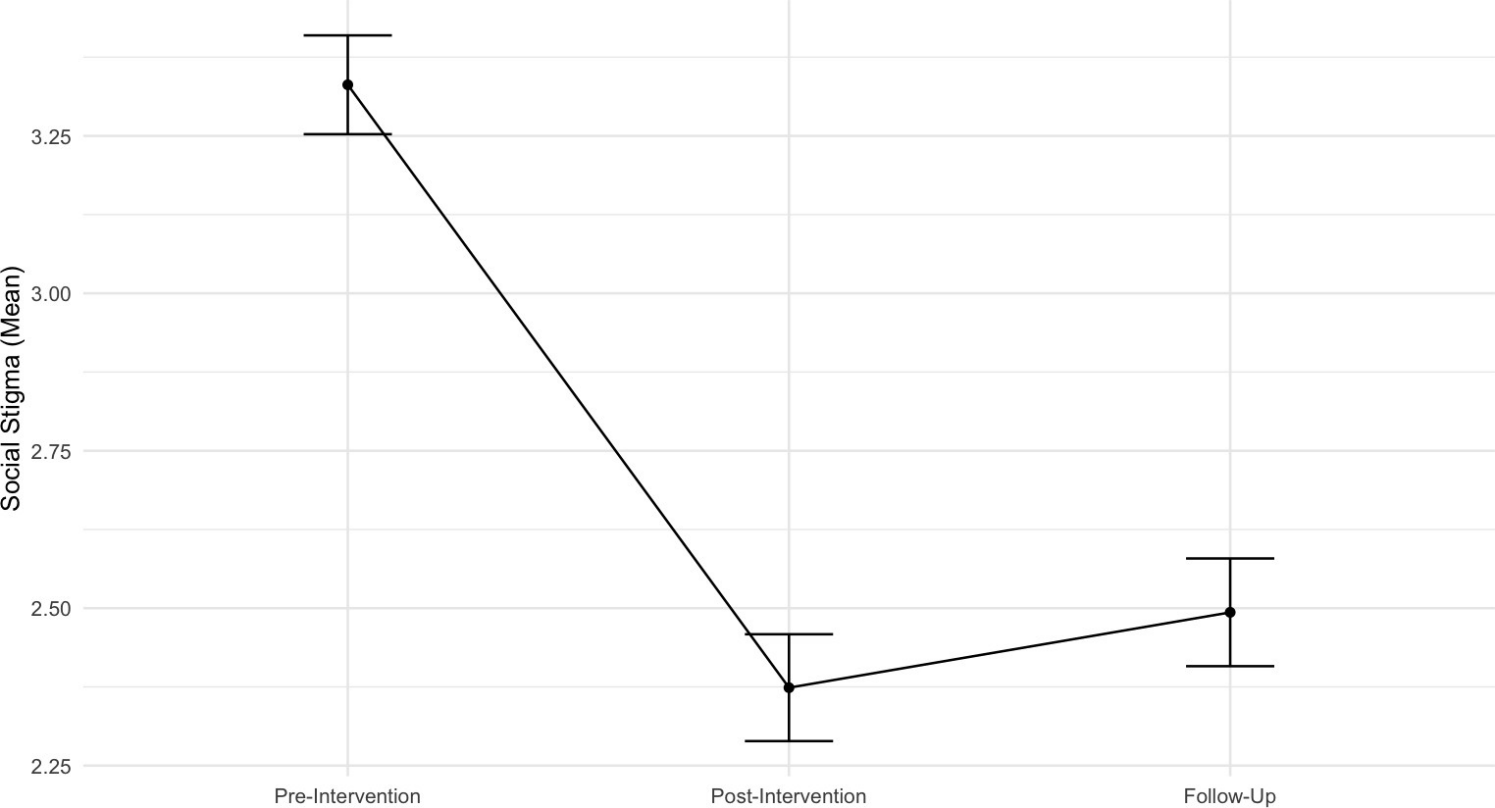
Mean standard errors across time points for the outcome of social stigma.

Results of the linear mixed effects model considering the evolution of social stigma across various time points show a significant negative effect of Time (*b* = − 0.479, SE = 0.05, t (214.38) = − 9.31, *p* < .001, 95% CI [− 0.58, − 0.38]), indicating that participants social stigma toward individuals with mental health problems decreased with time. Contrasts with Bonferroni correction showed a significant decrease in social stigma between the pre-intervention and post-intervention (*b* = - 0.96, *SE* = 0.078, *p* < .001), that persists until the follow-up moment (*b* = - 0.82, *SE* = 0.093, *p* < .001). There were no statistical differences between post-intervention and follow-up (*b* = 0.14, *SE* = 0.093, *p* = .42). Age (*b* = − 0.066, SE = 0.03, t (121.91) = − 1.93, *p* = .057, 95% CI [− 0.13, 0.001]) was marginally significant, showing that with age there is a tendency to face more anxiety about people with mental problems. The variable Previous psychological problems is not statistically significant (*b* = 0.249, SE = 0.21, t (121.49) = 1.21, *p* = .230, 95% CI [− 0.15, 0.65]). Fig 7 illustrates means and standard errors across time for this outcome.

The random intercept model indicated significant variability between individuals in their anxiety related to people with mental health issues (*p* < .001), with a variance component of 0.42 (*SD* = 0.65) and an ICC of 0.464, suggesting 46% of the variance was due to between-individual differences.

### Phase 2: Qualitative evaluation of the program

Table 5 presents the categories and subcategories for the program’s qualitative evaluation and the respective excerpts from the focus groups.

**Table 5:** Categories, subcategories, and examples of excerpts from the transcripts.

Categories	Subcategories	Excerpts
Positive aspects of the program	Psychoeducational sessions	*"The psychoeducational sessions were great*. *I remember one when we closed our eyes and breathed*.*" (male*, *12 years old)* *"(*.* *.* *.*) in the morning*, *when we were all together in psychoeducational sessions*, *it was also nice to see everyone in the class*, *and we always learned something*.*" (female*, *15 years old)*
	Arts sessions	*"There was an activity we did that also showed how music can have an influence on us and*, *well*, *it can change situations*.*" (female*, *15 years old)* *"I also really liked the cinema (*.* *.* *.*) I think it was cool that we learned how to edit*, *and I also learned a lot of things*.*" (female*, *15 years old)*
	Extra-session moments	*"We were always having breaks to have a snack and things like that*, *or to drink water*, *or go to the bathroom*.*" (female*, *14 years old)* *"*.* *.* *.*the breaks were also good for relaxing a bit*.*" (male*, *13 years old)*
	Logistical aspects	*"I thought the location was good and well situated too*.*" (male*, *13 years old)* *"I think it was good to be on vacation because we didn’t have to worry about school*.*" (female*, *15 years old)*
Negative aspects of the program	Schedule	*"The time each day*, *for me*, *was too long*.*" (male*, *12 years old)* *“The time*, *the length of the days could be a little shorter*.*” (male*, *13 years old)*
	Start time	*"I’ll just put it a bit later*.*" (female*, *15 years old)*
	Program duration	*"I think it could have been*.* *.* *. *like seven days*, *because that way we’d also have more time to get to know people better*, *and we’d have more time to talk about mental health*.*" (female*, *15 years old)* *“I think four days was not enough*.*” (female*, *14 years old)*
Opportunities for program implementation	Planning of the program	*"I think the program planning was good*, *so it made things easier*.*" (male*, *13 years old)* *"It was a perfect match and*, *when we did all four activities in a row*, *it all went well together*.*" (female*, *15 years old)*
	Program facilitators	*"The teachers and monitors interacted very well with us and communicated very effectively*.*" (female*, *15 years old)*
Benefits of the program	Learnings acquired	*"I think I understand more about what mental health is because I think I wasn’t aware of the things that happen and that there were so many mental health problems*.*" (male*, *13 years old)* *“I think it even helped us to open up more (*..*) and to become a little more extroverted*.*" (female*, *15 years old)*
	Change in attitudes	*"(*.* *.* *.*) I would try to help them get through it*, *to consult a psychiatrist*.*" (male*, *12 years old)* *"After the program*, *we gave more visibility to people who have problems*, *and then I think it improved my way of seeing people*.*" (female*, *15 years old)*
	Interpersonal relationships	*"I felt a bit lost; I didn’t know anyone*, *and then people welcomed me*.*" (female*, *12 years old)* *"(*.* *.* *.*) it was a chance to enjoy*, *make friends*, *and meet people*.*" (female*, *15 years old)*
Suggestions for program improvement	Examples for intervention	"*If someone has an anxiety crisis*, *how can we help without calling an adult*.*" (female*, *15 years old)*
	New activities	*"I would do*, *100% sure*, *an activity related to sports and mental health as well*.*" (male*, *13 years old)* *"(*.* *.* *.*) for example*, *a peddy-paper (*.* *.* *.*) So*, *people would get to know the place and also learn about mental health*.*" (female*, *15 years old)*
	Adaption to other age groups	*"People aged 65 or more*, *because there are many people in that age group who don’t understand*.*" (male*, *13 years old)* *"Sometimes it’s good for younger people to already understand what mental health is*.*" (female*, *15 years old)*

The content analysis results revealed five categories: positive aspects of the program, negative aspects, opportunities for program implementation, benefits, and suggestions for program improvement. The category of positive aspects of the program refers to the general characteristics and qualities that participants indicated as most positive in the program. Within this category, four subcategories were identified, namely: psychoeducational sessions, arts sessions, extra-session moments, and logistical aspects. Regarding psychoeducational sessions, the participants highlighted the implementation of practical activities that facilitated the approach and understanding of the topic of mental health. About the art sessions (cinema, theatre, music, and visual arts), the participants defined them as positive and cool, as well as a contribution to the association with emotions. Regarding extra-session moments, participants highlighted the breaks between sessions, which they considered beneficial for meeting some basic needs and relaxing. Finally, in the subcategory of logistical aspects, participants indicated that it was positive that the program was implemented during school breaks, as well as its location, facilities, and sequence of activities.

The category of negative aspects of the program refers to the general characteristics that participants indicated as least positive in the program, and three subcategories were identified: schedule, session start time, and program duration. Regarding the schedule, some participants reported that the schedule each day was long and could be reduced a little. Concerning the session start time, only one participant indicated that the sessions started early, preferring the start time to be later. Finally, in the subcategory of program duration, some participants said they would prefer the program to last longer than four days.

The category of opportunities for program implementation refers to aspects that facilitated or contributed to the program running smoothly, and two subcategories were identified: planning of the program and program facilitators. Regarding the planning of the program, participants named the combination of arts with psychology, the selected activities, and the way the program was organized. In the subcategory of program facilitators, participants reported that the facilitators eased communication among everyone, which contributed to the program running smoothly.

The category of benefits of the program refers to personal and social gains that participants take from their participation in the program, and three subcategories were identified: learnings acquired, change in attitudes towards people with mental health problems, and interpersonal relationships. Participants reported several key learnings from the program, notably an increase in their knowledge about mental health and the stigma surrounding it. In terms of attitude changes, they expressed that the program helped them develop a more positive perspective towards individuals with mental health issues, recognizing the importance of combating prejudice and promoting supportive, help-seeking behaviors. Additionally, participants noted that the program provided opportunities to meet new people and form new friendships, enhancing their interpersonal relationships.

The category of suggestions for program improvement refers to opinions and examples of aspects that could be improved or added in a future edition of the program, and three subcategories were identified: examples for intervention, new activities, and adaptation to other age groups. Regarding intervention examples, one of the participants suggested adding practical examples of how to help people with mental health problems in crisis and how to act in the absence of an adult. Concerning new activities, some participants mentioned that it would be interesting if, in addition to the arts, future intervention programs included activities such as sports, martial arts, and peddy-papers. Finally, in the subcategory of adaptation to other age groups, some participants suggested the development of similar intervention programs that would target other age groups, namely younger children and older adults.

## Discussion

This study aims to assess the impact of an arts-based program on participants’ mental health knowledge, social stigma, intergroup anxiety, and desire for social distance, its sustainability over time, and gather qualitative data to understand participants’ perspectives, experiences, satisfaction, and perceived benefits or drawbacks.

The findings regarding the improvement in participants’ **knowledge of mental health** issues provide compelling evidence of the program’s efficacy, with significant knowledge gains from pre- to post-intervention that were sustained at follow-up. Also, the lack of significant associations between age and previous psychological problems and knowledge improvement suggests that the program was effective across diverse demographic and psychological backgrounds. This aligns with previous research suggesting that psychoeducational interventions can effectively enhance understanding and awareness of mental health [52, 53]. The use of creative activities (e.g., music, visual arts, and theatre) in interventions consistently demonstrates improvements in participants’ mental health knowledge [54, 55], which is also coherent with our findings. Therefore, using arts to access mental health information may enable emotional connection and engagement of multiple senses and cognitive processes, making learning enjoyable and enhancing retention [56], which could foster a more profound understanding and challenge misconceptions and stereotypes about mental health.

Addressing **intergroup anxiety** is essential for reducing stigma and promoting positive attitudes towards individuals with mental illness [57]. We found a significant decrease in intergroup anxiety from pre- to post-intervention, maintained at follow-up. This suggests the program lessened anxiety, reflecting a shift in participants’ negative expectations and fears, which can hinder social interactions and perpetuate stereotypes, prejudice [58], and negative attitudes [57]. This aligns with research showing that interventions aimed at reducing intergroup anxiety can promote positive mental health schema among children [59]. Our results also align with a national study that showed a positive impact of an intervention targeting stigma reduction in reducing intergroup anxiety and negative attitudes towards individuals with mental illness [60]. However, the impact of such interventions may vary by age and gender, with girls exhibiting increased intergroup anxiety as they get older [60]. This is also coherent with our study, which showed a significant positive association between age and intergroup anxiety. Older participants may have unique experiences or perspectives, highlighting the importance of tailoring interventions to different age groups within youth. Additionally, the finding that participants without a psychological problem exhibited higher levels of intergroup anxiety underscores the pervasive nature of stigma and anxiety, even among individuals who may not have personal experiences with mental health issues [61].

Similarly to intergroup anxiety, the findings regarding **social distance** revealed a decrease in participants’ desire to avoid interaction with people with mental health problems from pre- to post-intervention. These findings may be related to the association that has been suggested between more knowledge about mental health problems and lower social distance [62]. According to the path model [63], people who are familiar with mental health problems are less likely to have negative beliefs and fear of interacting with people with mental health problems, which leads to less social distance and, consequently, to less stigmatizing attitudes. Therefore, a possible explanation for our findings concerns the psychoeducational sessions of the program that have increased participants’ knowledge about mental health and the consequences of mental health-related stigma, which consequently decreased the desire for social distance towards people with mental health problems.

The study also demonstrated that the program significantly reduced **social stigma** immediately after the intervention and, even more impressively, six months later, in line with previous interventions that included psychoeducational content, social contact, and arts activities [17, 27]. This underscores the long-lasting effectiveness of utilizing art for social change. Through creative expression and engagement with various art forms, participants gained a deeper understanding of social issues and developed empathy and respect for diverse perspectives. These transformative experiences cultivated a more inclusive and accepting environment beyond the program’s conclusion, highlighting the potential of arts-based interventions to instill lasting positive attitudes and behaviors in young individuals [27].

The quantitative findings provided a deeper understanding of the positive impact of the intervention. Participants highlighted the psychoeducational and arts sessions for enhancing individual and social dimensions, improving personal skills (e.g., communication and identification of emotions), increasing knowledge about mental health and mental health-related stigma, and changing attitudes towards people with mental health problems. Additionally, they positively perceived the program, indicating aspects such as organization, activities, facilitators, location, and implementation period, as well as breaks and mealtimes. Regarding areas of improvement, participants voiced concerns about the time and duration of the sessions per day, suggesting a later start in the morning and a reduction of the hours per day.

Nonetheless, the desire for more sessions and a longer intervention duration reflects their positive engagement and satisfaction with the program, underscoring their enthusiasm for continued involvement. Lastly, participants’ recommendations for future editions, such as delving deeper into addressing specific mental health issues and adapting the program to different age groups, illuminate their keen interest and willingness to continue learning in the mental health field. Their eagerness to provide ideas for future sessions reflects their deep curiosity about mental health and their motivation to explore and engage with these critical issues.

### Limitations and recommendations for future research

Despite the insights gained from this study, it is essential to recognize and address its limitations. Although a longitudinal study has its advantages, such as flexibility in study design and the ability to capture individual changes, the absence of a control group brings certain limitations to the study, and the results should be interpreted with caution regarding causality and generalization. Therefore, without a control group for direct comparison, it becomes challenging to determine whether the observed changes over time are truly due to the variable of interest or influenced by other unaccounted factors. Moreover, without a control group, it may be difficult to differentiate between actual changes over time and the typical maturation effects that naturally happen with aging or development. Future research should include control groups to address these limitations and provide more substantial evidence for the effectiveness of the intervention. Second, common and specific mechanisms of change were not controlled or measured. Future studies should measure and analyze mediators of change, identifying the underlying processes responsible for stigma reduction. Third, although we have included participants with mental health problems, they were a minority group, which limited the possibility of analyzing the impact of the program on internalized stigma. Plus, these problems were self-identified, lacking a rigorous method of confirming the authenticity of clinical information. Future research should include larger groups of participants with mental health issues. Fourth, the response rate in the follow-up was considerably smaller, which limited the interpretation and generalizability of these results.

### Contributions of the study

The present study has several positive contributions that must be highlighted. Working with younger groups provides an opportunity to promote stigma prevention and reduction for future generations. The pre-validation with scientific and technical experts (stakeholders, members of ONGs, and experts) enhanced its quality and suitability. The multimodal approach, combined with participatory creative techniques, literacy, and psychoeducational tools, was an innovative and CYP-friendly way to address this sensitive topic. The program used an indirect and ethical approach to social contact. by joining participants with and without mental health problems, without identifying them, and aligning effective practices from previous research [17]. The four-day immersive experience and random sampling with an intervention tailored for a specific group improved the study’s quality, while the six-month follow-up provided evidence of the program’s lasting impact. The program also had clear procedures and a toolkit that would soon be available to the public. The study employed validated and appropriate outcome measures to assess the primary outcomes used an adjusted sample size, and included follow-up data collection, enabling the consistency of results to be tested over time, as recommended in a narrative synthesis of systematic reviews [64]. The longitudinal mixed-methods design provided not only a long-term evaluation of the program’s impact but also detailed process analyses based on the participants’ perceptions, complementing the quantitative data derived from the survey.

### Implications of the study

The study offers several practical implications, including the potential to generalize and adapt our program to various contexts and populations. It highlights the importance of early intervention strategies to prevent issues related to discrimination, the value of collaboration with artists to enhance mental health literacy, and the role of policy advocacy through participative, child-friendly interventions. Additionally, it emphasizes the need for community engagement and ongoing efforts in evaluation and improvement. In particular, the validation of this program suggests that it can be an effective tool for reducing mental health stigma among youth. Organizations, schools, and mental health facilities can implement similar programs based on this validated model. It can encourage practitioners to incorporate arts-based elements into their clinical, preventive, and psychoeducational interventions, involving art, music, drama, or other creative forms to engage youth in discussions about mental health, making the sessions more appealing and effective [65]. Since the program targets youth, it emphasizes the importance of early intervention in addressing mental health stigma [66]. Schools and youth organizations can use this study to justify the need for such interventions at an early age to prevent stigma from becoming deeply ingrained [67]. The findings highlight the importance of psychoeducation in reducing stigma [68]. Therefore, practitioners can focus on educating youth about mental health and the impact of stigma, empowering them to recognize, increase awareness, and challenge stigmatizing attitudes and behaviors [17].

It also encourages collaborations between mental health professionals and artists that can play a crucial role in creating materials and activities that resonate with youth, making mental health education more engaging and impactful [69]. The validated program can be used as a basis for community engagement initiatives, workshops, events, or campaigns aimed at reducing mental health stigma among youth [10]. Evidence-based interventions should be endorsed and embedded within national policies and strategies to ensure resources are used effectively and mental health outcomes are enhanced. Promoting these programs is crucial for obtaining the necessary funding and backing from policymakers, which can facilitate their extensive implementation in schools and communities, thereby contributing to broader discussions on mental health policy [70]. Furthermore, the practitioners can learn from the study’s methodology and evaluation techniques to continuously assess and improve their programs. Regular evaluation helps ensure effectiveness and allows for adjustments based on feedback and outcomes. If the study could be conducted in a specific cultural, ethnic, social, and clinical context, practitioners could consider adapting and assessing the program’s effectiveness to fit different backgrounds (e.g., adolescents with psychological problems). Plus, professionals working with youth, such as teachers, counselors, and youth workers, can benefit from training based on the validated program, equipping them with the knowledge and skills to implement similar interventions effectively [71].

## Conclusion

This study has provided valuable insights about the impact of an innovative arts-based program to reduce mental health-related stigma, underscoring the importance of holistic and creative strategies in our efforts to promote tolerant attitudes and reduce stigma among youth. Through rigorous assessment and analysis, the program significantly improved attitudes toward mental health, reduced stigma and intergroup anxiety, and promoted evidence-based knowledge. Combining creative and expressive activities with scientific CYP-friendly psychoeducation, participants gained a deeper understanding of mental health issues and developed greater empathy and acceptance. The program successfully created a safe and supportive environment for open dialogue about mental health. Therefore, it underscores the importance of continuing to invest in initiatives that create a positive supportive environment for those facing mental health challenges [7, 72], as well as in the ongoing evaluation and enhancement of such programs to ensure their continued efficacy and effectiveness in combating stigma and fostering mental well-being among our youth [73]. By investing in such novel approaches, we can continue to break down barriers, challenge stereotypes, and create a more inclusive society for individuals living with mental health conditions.
